# LRTae: improving statistical power for genetic association with case/control data when phenotype and/or genotype misclassification errors are present

**DOI:** 10.1186/1471-2156-7-24

**Published:** 2006-04-27

**Authors:** Sandra Barral, Chad Haynes, Millicent Stone, Derek Gordon

**Affiliations:** 1 Laboratory of Statistical Genetics, Rockefeller University, New York, USA; 2 Department of Medicine, University of Toronto, Toronto, Canada; 3 Department of Genetics, Rutgers University, Piscataway, USA

## Abstract

**Background:**

In the field of statistical genetics, phenotype and genotype misclassification errors can substantially reduce power to detect association with genetic case/control studies. Misclassification also can bias population frequency parameters such as genotype, haplotype, or multi-locus genotype frequencies. These problems are of particular concern in case/control designs because, short of repeated sampling, there is no way to detect misclassification errors.

We developed a double-sampling procedure for case/control genetic association using a likelihood ratio test framework. Different approaches have been proposed to deal with misclassification errors. We have chosen the likelihood framework because of the ease with which misclassification probabilities may be incorporated into in the statistical framework and hypothesis testing. The statistic is called the Likelihood Ratio Test allowing for errors (LRTae) and is freely available via software download.

**Results:**

We applied our procedure to 10,000 replicates of simulated case/control data in which we introduced phenotype misclassification errors. The phenotype considered is Ankylosing Spondylitis (AS). The LRTae method power was always greater than LRTstd power for the significance levels considered (5%, 1%, 0.1%, 0.01%). Power gains for the LRTae method over the LRTstd method increased as the significance level became more stringent. Multi-locus genotype frequency estimates using LRTae method were more accurate than estimates using LRTstd method.

**Conclusion:**

The LRTae method can be applied to single-locus genotypes, multi-locus genotypes, or multi-locus haplotypes in a case/control framework and can be more powerful to detect association in case/control studies when both genotype and/or phenotype errors are present. Furthermore, the LRTae method provides asymptotically unbiased estimates of case and control genotype frequencies, as well as estimates of phenotype and/or genotype misclassification rates.

## Background

In the field of statistical genetics, phenotype and genotype misclassification errors can substantially reduce power to detect association with genetic case/control studies [1-4]. Misclassification also can bias population frequency parameters such as genotype, haplotype, or multi-locus genotype frequencies. These problems are of particular concern in case/control designs because, short of repeated sampling, there is no way to detect misclassification errors [5].

We developed a double-sampling procedure for case/control genetic association using a likelihood ratio test framework [6]. Different approaches have been proposed to deal with misclassification errors [7]. We chose the likelihood framework because of the ease with which misclassification probabilities may be incorporated into in the statistical framework and hypothesis testing.

The statistic is called the Likelihood Ratio Test allowing for errors (LRTae) and is freely available via software download [8]. We applied our procedure to 10,000 replicates of simulated case/control data in which we introduced phenotype misclassification errors. The phenotype considered is Ankylosing Spondylitis (AS).

## Implementation

The program is compiled to work in UNIX Solaris, LINUX, and Windows (PC) operating systems. All commands are executed from the command line in UNIX and LINUX or DOS prompt in Windows. A full description of file requirements and program features is available via the web [9].

## Results

We simulated 10,000 replicates of case/control genetic association data to evaluate power and estimation of multi-locus genotypes frequencies for the LRTae and LRTstd (the likelihood ratio test that does not include double-sample information) methods. By double-sample we mean that some individuals are measured twice: once with the standard measurement instrument (called the fallible measurement) and once with a gold-standard measurement instrument (called the infallible measurement) [10,11].

Generating multi-locus genotype frequencies for cases and controls were determined using real data for 3 SNPs (TNF308, TNF863, and TNF1031) in the promoter region of the TNF-alpha locus from cases and controls ascertained for an actual Ankylosing Spondylitis (AS) association study. More specifically, we considered a Northern European ancestry Caucasian sample population consisting of 169 individuals affected with AS and 202 unaffected individuals who were matched on age, gender, and ethnicity. AS was diagnosed according to the modified New York Criteria that requires patients to have bilateral sacroiliitis on plain X-ray [12]. The generating frequencies are presented in Table 1.

**Table 1:** Generating multi-locus genotype frequencies for simulations

Multi-locus genotypes	0	1	2	3	4	5	6	7	8	9	10	11	12
Case	0.00	0.02	0.00	0.01	0.01	0.00	0.20	0.00	0.02	0.12	0.00	0.06	0.58
Control	0.01	0.04	0.01	0.05	0.00	0.03	0.22	0.03	0.03	0.20	0.01	0.05	0.33

Legend for Table 1. In this table we provide the generating frequencies in case and control populations for the thirteen multi-locus genotypes. We number the genotype frequencies 0 – 12. The multi-locus genotype corresponding to each coded genotype (0–12) is given under the heading "Genotype Codings" (see directly below). Note that, at each SNP, we use the code 1, 2, 3 to refer to the less common homozygote, heterozygote, and more common homozygote, respectively. For example, the code "3 : 3 : 3" is the multi-locus genotype consisting of the more common homozygote at each of the three SNP loci. Also note that these generating frequencies suggest a recessive mode of inheritance for AS in our simulations, since greatest risk occurs for individuals who are homozygous for each of the three SNPs (code 3 : 3 : 3).

Genotype Codings:

0 = 1 : 2 : 2

1 = 1 : 3 : 3

2 = 2 : 1 : 1

3 = 2 : 2 : 2

4 = 2 : 2 : 3

5 = 2 : 3 : 2

6 = 2 : 3 : 3

7 = 3 : 1 : 1

8 = 3 : 2 : 1

9 = 3 : 2 : 2

10 = 3 : 3 : 1

11 = 3 : 3 : 2

12 = 3 : 3 : 3

We set generating phenotype misclassification rates in each direction (case→ control, control→ case) at 10%. We did not generate genotype misclassification errors in these data because of limitations in determining permutation p-values when both categories are double-sampled. For each replicate, we randomly sampled 25% of the individuals to obtain infallible phenotype measurements. To estimate power for the LRTae and LRTstd methods, we computed permutation p-values. For each replicate, we computed the permutation p-value by randomly reassigning the multi-locus genotypes for all individuals, keeping the total count of each multi-locus genotype classification fixed. The proportion of permuted data sets whose LRTae or LRTstd statistic exceeds the observed dataset statistic is the permutation p-value for the corresponding statistic. We computed power by determining the proportion of replicates for which permutation p-values exceeded given thresholds.

Our results for permutation power are presented in Table 2. In this table, we observe that at each significance level (0.05, 0.01, 0.001, 0.0001), the LRTae power is always greater than the LRTstd power. Furthermore, the power difference (LRTae power – LRTstd power) increases as the significance level becomes more stringent. For example, the minimum power difference of about 0.02 occurs for the 0.05 significance level and the maximum power difference of about 0.15 occurs for the most stringent significance level of 0.0001.

**Table 2:** Power for LRTae and LRTstd methods using permutation p-values

*Sgn Level*	*Power*		*Power Difference*
		
	LRTae	LRTstd	
0.05	0.989	0.970	0.019
0.01	0.953	0.905	0.048
0.001	0.831	0.716	0.115
0.0001	0.677	0.532	0.145

Legend for Table 2. In this table, we report the proportion of replicates (out of 10,000) whose permutation p-values for each method (LRTae, LRTstd) are less than the Significance Level (Column labeled Sgn Level) threshold. The thresholds we consider are 0.05, 0.01, 0.001, and 0.0001. We also report the Power Difference (Power(LRTae) – Power(LRTstd)) for each significance level.

We also comment that the LRTae mean estimates of multi-locus genotype frequencies in cases and controls and misclassification error rates were very accurate (data not shown) with relatively small variances.

## Conclusion

One of the main concerns for case/control genetic association studies is the presence of undetectable genotype and phenotype errors, since these misclassifications may lead to a loss in statistical power to detect association and may also lead to incorrect estimation of parameters such as multi-locus genotype- or haplotype- frequencies in cases and controls [1,13,14].

Although statistical methods incorporating genetic models of inheritance in association studies have verified potential power gains [15-18], we did not specify any genetic inheritance model for AS in our study since there is no consistent mode of inheritance suggested for AS [19]. We note however that dominant and recessive modes have been reported for particular data sets [20,21].

Regarding the impact of these misclassification errors on false positive rates (or Type I error), when non-differential misclassification is considered (that is, the misclassification rates are equal in the cross-classified groups), there is no increase in Type I error [1,4,13,22]. In the case of differential misclassification, researchers [23] recently demonstrated in a diabetes association study that there is an increase in the Type I error rates when case and control groups have differential genotype error rates.

The observed increase in statistical power of the LTRae statistic is due the presence of double sample on some individuals; the infallible measurement establishes the correct classification for those individuals. Furthermore, the misclassification rates that are estimated from the double-sample data are used to "weight" phenotype classifications for the entire sample. More details are provided in our previous work [6]. We note that, if we have correct misclassification rates but no double-sample information, our LRTae method is not necessarily more powerful than the LRTstd method [6]. Double-sampling methods are among the few methods, along with Bayesian methods [7], that enable researches to detect and treat misclassification errors.

We also performed simulations using double-sampling proportions of 50% and 10% (full results not shown). As noted above, power gains for the LRTae method (Table 2) increased as the double-sampling proportion increased. However, in all simulations, LRTae mean estimates of multi-locus genotype frequencies in cases and controls and misclassification error rates were accurate. For the interested reader, we comment that we recently published the non-centrality parameter for our LRTae method [24]. This parameter enables researchers to perform power and sample size calculations for the LRTae statistic and also to perform cost/benefits analyses. That is, researchers may ask whether, for a fixed cost, it is more powerful to genotype larger samples subject to misclassification error or to collect smaller samples, some proportion of which have been double-sampled.

The LRTae method has the flexibility to perform case/control association analysis when the genotype data are single-locus genotypes, multi-locus genotypes, or multi-locus haplotypes, and to determine significance via permutation methods when double-sampling has been performed on only one of the categories (phenotype or genotype).

When double sampling data are available on either phenotypes, genotypes, and/or haplotypes, we strongly recommended the LRTae method to: (i) improve the statistical power to detect genetic association in case/control studies in the presence of misclassification error; (ii) obtain estimates of misclassification probabilities from double sample information; and (iii) use these misclassification parameters to weight the estimates of population frequency parameters.

## Availability and requirements

Project name: LRTae software.

Project home page: See [9].

Software availability: See [8].

Operating system: UNIX Solaris, LINUX, and Windows.

Programming language: C++.

Other requirements: None.

License: None.

Any restrictions to use by non-academics: None.

The software is freely available via download from the ftp site listed above (Software availability).

## Abbreviations

LRTae- Likelihood Ratio Test allowing for errors

LRTstd- Likelihood Ratio Test standard method

SNP- Single Nucleotide Polymorphism

AS- Ankylosing Spondylitis

TNF- Tumor Necrosis Factor

## Competing interests

The author(s) declare that they have no competing interests.

## Authors' contributions

Sandra Barral performed the statistical analyses and wrote the majority of the manuscript. Chad Haynes developed the software. Millicent Stone and Derek Gordon conceived of the study, participated in its design and coordination, and helped to draft the manuscript.
